# New evidence for the sensorimotor mismatch theory of weight perception and the size-weight illusion

**DOI:** 10.1007/s00221-024-06849-0

**Published:** 2024-05-23

**Authors:** Jarrod W. C. Harris, Elizabeth J. Saccone, Rebecca Chong, Gavin Buckingham, Melanie J. Murphy, Philippe A. Chouinard

**Affiliations:** 1 Department of Psychology, Counselling, and Therapy, School of Psychology and Public Health, La Trobe University, George Singer Building, Room 460, La Trobe University, Bundoora Campus, Melbourne, VIC 3086 Australia; 2 Department of Psychological and Brain Sciences, Johns Hopkins University, Baltimore, MD USA; 3 Sport and Health Sciences, College of Life and Environmental Sciences, University of Exeter, Exeter, UK

**Keywords:** Weight perception, Size-weight illusion, Sensorimotor mismatch theory, Lifting forces, Principal components analysis

## Abstract

The size-weight illusion is a phenomenon where a smaller object is perceived heavier than an equally weighted larger object. The sensorimotor mismatch theory proposed that this illusion occurs because of a mismatch between efferent motor commands and afferent sensory feedback received when lifting large and small objects (i.e., the application of too little and too much lifting force, respectively). This explanation has been undermined by studies demonstrating a separation between the perceived weight of objects and the lifting forces that are applied on them. However, this research suffers from inconsistencies in the choice of lifting force measures reported. Therefore, we examined the contribution of sensorimotor mismatch in the perception of weight in the size-weight illusion and in non-size-weight illusion stimuli and evaluated the use of a lifting force aggregate measure comprising the four most common lifting force measures used in previous research. In doing so, the sensorimotor mismatch theory was mostly supported. In a size-weight illusion experiment, the lifting forces correlated with weight perception and, contrary to some earlier research, did not adapt over time. In a non-size-weight illusion experiment, switches between lifting light and heavy objects resulted in perceiving the weight of these objects differently compared to no switch trials, which mirrored differences in the manner participants applied forces on the objects. Additionally, we reveal that our force aggregate measure can allow for a more sensitive and objective examination of the effects of lifting forces on objects.

**Supplementary Information:**

The online version contains supplementary material available at 10.1007/s00221-024-06849-0.

## Introduction

The size-weight illusion (SWI) occurs when the smaller of two equally weighted objects is perceived heavier (Charpentier 1886, 1891). One explanation, known as the sensorimotor mismatch theory (SMT), contends that our perception of an object’s weight relates to the forces we apply to lift it (Davis and Roberts 1976; Granit 1972; Gregory 1968; Müller and Schumann 1889; see also: Murray et al. 1999). In other words, the forces utilised to lift objects influence their perceived weight. In the case of the SWI, participants typically expect the smaller object to be lighter, leading to an initial insufficient application of force. Consequently, the lift is slower and more difficult than anticipated, resulting in the object being perceived heavier. Conversely, when lifting the larger object, people tend to initially apply excessive force, and the object is subsequently lifted more quickly than expected, which results in a decrease in perceived weight.

Expectations of weight, and the degree to which we apply forces on objects, may or may not occur with us consciously aware of them. The same applies to the influence that a mismatch in sensory feedback about an object’s weight can have on our perception of its weight. Indeed, we do not think it would be a leap to say that these processes frequently occur outside conscious awareness. It then follows that SMT could depend on mechanisms that are not cognitively demanding. For this reason, we agnostically treat SMT as a theory that may or may not necessitate cognitive processing, defined as any operation related to conscious intellectual activity. Furthermore, we will use the terms *expectations of weight* and *sensorimotor predictions* interchangeably to mean more or less the same thing. Of course, we may become consciously aware of a mismatch when it is unusually large, but this awareness conceivably occurs from directing attention to the discrepancy rather than being the central driver of weight perception—in the same way we become aware of traffic signals after initiating automatic responses to them.

The relationship between effort and weight perception, as predicted by SMT, has been investigated in experiments that varied the manner participants lifted objects. For example, Davis (1973) had participants lift equally weighted large and small objects with arm movements pivoting from the elbow. A typical SWI was found when both objects were lifted in the same manner. In contrast, when the large object was placed further away from the elbow than the smaller one, requiring more force to the lift the latter, the illusion diminished markedly.

Later, Davis et al. (1977) demonstrated that the perceived weight of an object can decrease if participants are instructed to lift it vigorously (i.e., more forcefully) compared to gently (i.e., less forcefully). As predicted by SMT, both studies demonstrate that a given object can feel lighter or heavier depending on the effort used to lift it.

Other studies measured the height and acceleration of an object being lifted to determine if these factors might influence weight perception (Davis and Roberts 1976; Loomis 1907). Davis and Roberts (1976) used video recordings to measure the maximum height participants lifted small and large cans of equal mass, as well as the time taken to reach these heights (i.e., peak acceleration). The results supported SMT. The authors found that the large can was often lifted more quickly and perceived lighter—even though both objects were lifted to the same height. Equally notable, the higher acceleration for the large can was only present on trials when participants reported experiencing the SWI. For trials where there was no difference in acceleration, there was also no perceived difference in weight.

However, SMT fell out of favour as an explanation for the SWI after the publication of Flanagan and Beltzner’s (2000) study. The study had participants lift SWI objects with force transducer handles, which measured grip force (i.e., forces applied horizontally between the thumb and index finger), load force (i.e., forces applied vertically on the object), and the rates at which these forces were applied. The initial force rates to lift the objects were considered to reflect sensorimotor predictions, whereby a greater initial force indicated a greater expectation of an object’s weight. From the start to the end of 40 trials, the participants’ experience of the illusion remained constant—always perceiving the small object heavier than the larger one. In the initial trials, participants applied force rates that were higher and lower than required for the normal lifting of the large and small objects, respectively. However, in subsequent trials, the motor system learned to adapt to the actual mass of the object. There was no longer a discernible difference between the rates of force applied to either object—even though the SWI persisted. The central premise of SMT was therefore undermined. Perception of the illusory weight seemed to be unrelated to how participants applied forces. Flanagan and Beltzner’s (2000) findings were subsequently replicated multiple times, bolstering the case for a dissociation between the perceptual experience of the illusion and motor output (for example, Buckingham and Goodale 2010b; Chouinard et al. 2009; Grandy and Westwood 2006; Li et al. 2007; Rabe et al. 2009).

However, there is a growing number of studies that either do not replicate this adaptation (Buckingham and Goodale 2010a; Platkiewicz and Hayward 2014; Saccone et al. 2019a) or show incomplete adaptation (Buckingham and Goodale 2013; Li et al. 2011; Rabe et al. 2009) on some force measures. Rather, in these instances, some force measures demonstrated a persistent difference. For example, Li et al. (2011) found an interaction between object size (large, small) and trial (1–8) for peak grip force and peak grip force rate in which the difference in forces applied to the large and small objects diminished with trials. However, this same interaction was not found for peak load force rates, indicating that over the same number of trials, participants consistently applied more force to the large compared to the small object. Rabe et al. (2009) found a similar pattern of results over 16 trials. When motor adaptation is incomplete, it could be the case that the persistent *erroneous* application of force in the unadapted force measures constitute sufficient information for the sensorimotor system to detect a discrepancy between expected and experienced weight in an SMT manner, which can contribute to the SWI.

**Fig. 1:**
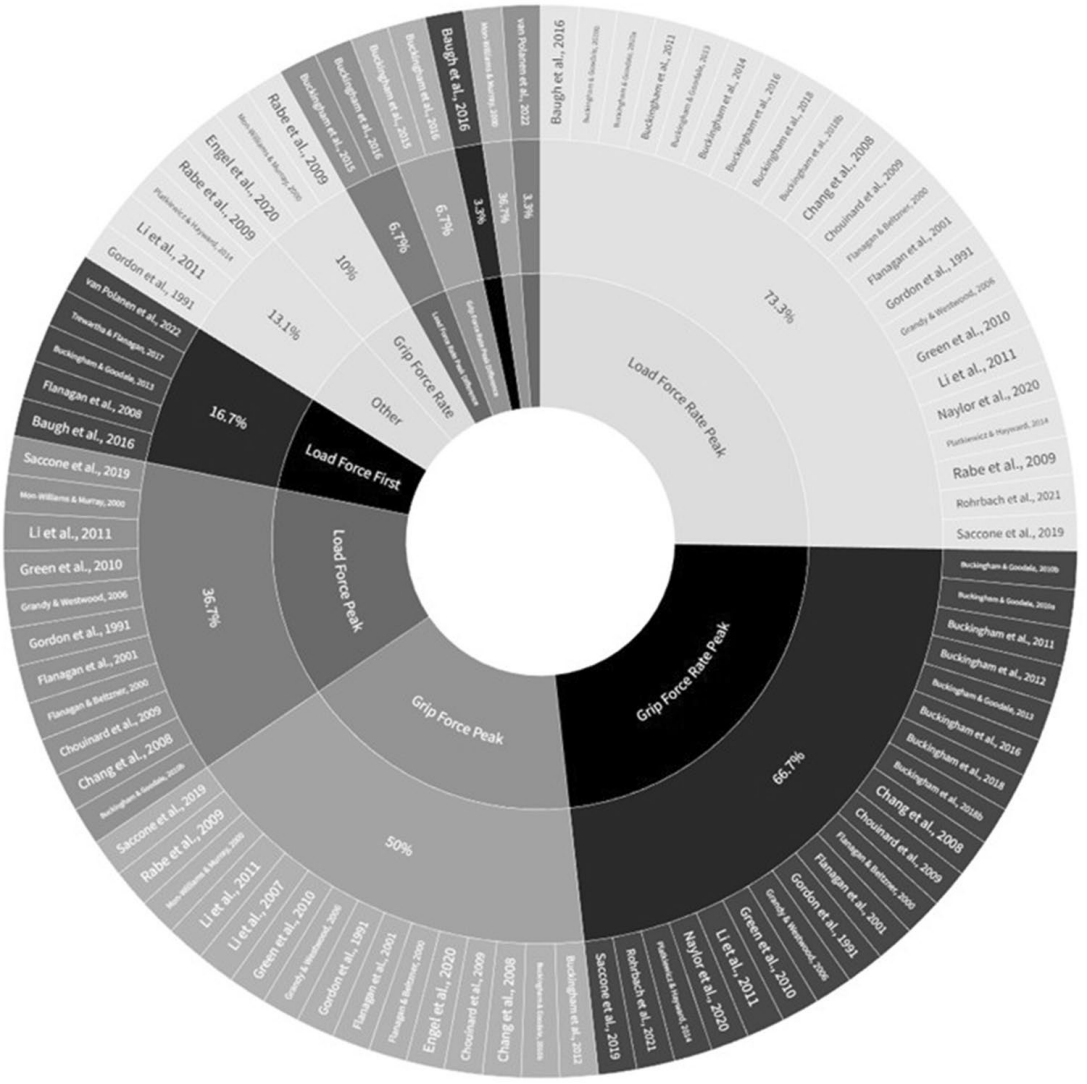
Summative representation of force measures reported in past research. A total of 30 papers are included in the figure using 11+ different measures. The most widely reported measures are: load force rate peak (73.3%), grip force rate peak (66.7%), grip force peak (50%), load force peak (36.7%). The remaining measures are as follows: load force at first peak in load force rate (16.7%); ‘other’ which includes vertical acceleration, vertical acceleration peak, and time-course of load force (13.3%); grip force rate (10%); load force rate peak difference (6.7%); grip force rate peak difference (6.7%); load force (3.3%); load force rate (3.3%); and grip force at first peak in grip force rate (3.3%). A table with this information can also be found in “Appendix A”

Discrepancies in motor adaptation findings seems exacerbated by the choice in force measures reported in papers. Dijker (2014) notes how it is unclear whether different force measures are equally representative of weight expectations and which force measures are most critical for ruling out SMT when they do adapt in SWI experiments. Yet, researchers report different force measures in different papers as depicted in Fig. 1, which encompasses 30 individual papers that measured forces in SWI experiments. These papers were selected through a non-exhaustive search of databases such as PubMed and PsycInfo using a combination of keywords related to ‘size-weight illusion’ and ‘force’. Note this summative representation is not a systematic review, which requires a more focused and rigorous investigation that is beyond the scope of this study. Nonetheless, it is clear from this simple exercise that there is no consensus as to which of the 11 + measures, and their combinations, are better suited to reflect expectations of weight. This is an important issue for assessing the merits of SMT for explaining the SWI. If no consensus exists on which force measures best reflect weight expectations, then it is difficult to be confident in claims against SMT based on previous SWI research demonstrating motor adaptation.

Compounding to the problem is the lack of justification and inconsistent justifications for choosing some force measures over others. Most papers do not specify the reasons for choosing the measures that they report (e.g., Buckingham et al. 2016; Flanagan et al. 2008; Naylor et al. 2020; Rabe et al. 2009). Only a minority of papers articulate why they chose to focus on particular force measures (Buckingham et al. 2012, 2015; Flanagan and Beltzner 2000; Li et al. 2011) or include data from non-reported force measures in the supplementary material (Chouinard et al. 2009; Saccone et al. 2019a). Without proper justification, force measure selection appears arbitrary, which does not relieve concerns of possible post-hoc selection. It could be the case that non-reported force measures did support SMT and that authors may have felt pressured not to report them given the influence of the Flanagan and Beltzner (2000) paper and contemporary thinking at the time of publication.

However, more recent studies demonstrate how variations in motor commands for lifting the same object can produce changes in its perceived weight (e.g., Maiello et al. 2018; van Polanen and Davare 2015). These studies had participants lift objects whose mass changed, sometimes without them knowing, so that they would lift them using forces better suited for the mass they had lifted in the previous trial as a result of either *sensorimotor memory*^1^ (Chouinard et al. 2005; Johansson and Westling 1988; van Polanen and Davare 2015) or having quickly learned to associate a mass to a particular visually presented object. Under either scenario, SMT predicts that the change in mass of an object will influence weight perception because the initial forces applied during lifting will either be too little or too great than what is normally applied for its real mass.

In one study, van Polanen and Davare (2015) had participants lift identical-looking light (220 g) and heavy (620 g) cubes with force transducers. The authors compared forces applied when participants lifted the same cube presented consecutively (i.e., light-after-light or heavy-after-heavy) to when the cubes had been switched (i.e., light-after-heavy or heavy-after-light). They also asked participants to report perceptual weight estimates after each lift to determine if changes in the force output might influence perception. For the light cube, the peak rates of grip and load force were greater on trials where there had been a switch (light-after-heavy) compared to when there was no switch (light-after-light). Consistent with SMT, participants perceived the light cube lighter for switch (light-after-heavy) than no-switch (light-after-light) trials. That is, the same light cube felt lighter when more force was applied to lift it as a result of having previously lifted the heavy cube.

In a different study, Maiello et al. (2018) had participants lift identical-looking light (50 g) and heavy (670 g) objects in an ABA sequence (i.e., heavy-light-heavy or light-heavy-light). Participants reported the light object as 40% lighter when it followed the heavy object—even though they were aware that they were lifting the same object as before. The opposite effect extended to the heavy object, which was perceived 13% heavier when it was lifted after the light object compared to when it was lifted initially. Although the authors did not measure lifting forces, we know from other studies that they are affected by changes in mass (Chouinard et al. 2005; van Polanen and Davare 2015), which could lead to changes in perceived weight according to SMT.

Considering these issues, we aimed to re-examine the merits of SMT by examining the influence of lifting forces on weight perception using a novel approach. We designed two experiments where the expectations of weight were driven by what participants lifted in a previous trial in experiment 1 and object size in experiment 2. In experiment 1, we had participants lift two objects that were identical in appearance but had different masses. In experiment 2, we had participants lift two objects that differed in size but had the same mass in a SWI paradigm. In both experiments, we measured grip and load forces. We performed a principal components analysis (PCA) on their peaks and peak rates, which our literature review revealed to be the four most commonly reported force measures (Fig. 1). This was done to determine whether or not they might reflect a common underlying process. The results from this analysis indicated that they did. For this reason, we calculated an aggregate measure of force across the four measures. This aggregate is arguably advantageous for evaluating SMT for the following four reasons. First, it is a more comprehensive choice because it encapsulates a broad range of forces into one measure. Second, it is a more precise choice given it increases the signal-to-noise ratio. Third, it is a more objective choice given that it does not appeal to rationalism, which can differ substantially among well-meaning researchers. Fourth, it is a choice that does not require one to statistically correct for multiple measures—relieving concerns of possible Type 1 errors. In the interests of transparency and comparison with past research, we also report peak grip and load forces and their rates.

We hypothesised that SMT would be supported by several lines of evidence demonstrating an influence of lifting force on weight perception. They are as follows. For experiment 1, we hypothesised that there would be greater lifting force and lower perceptual estimates of weight for the light object on trials following a switch (light-after-heavy) compared to trials where there was no switch (light-after-light). We also hypothesised a mirror reversal of effects for the heavy object where there would be lower lifting force and higher perceptual estimates of weight on trials following a switch (heavy-after-light) compared to trials where there was no switch (heavy-after-heavy). For experiment 2, we hypothesised greater lifting force and lower perceptual estimates of weight for the large object. Importantly, and contrary to the prevailing prediction, we further hypothesised that these differences would be sustained, at least to some extent. For both experiments, we also hypothesised that perceptual estimates of weight would correlate with lifting forces. Most of these specific hypothesise were supported—providing substantial support for SMT.

## Method

### Participants

Thirty-four volunteers (24 females, 10 males, mean age = 24.11 years, *SD* = 5.59) participated in the two experiments. Participants were required to be right-handed and have normal or corrected-to-normal vision. All participants gave written, informed consent to participate and received a gift voucher as compensation for their time. All study procedures were approved by the La Trobe University Human Research Ethics Committee and were conducted in accordance with the 1964 Helsinki Declaration.

### Stimuli

#### Practice stimuli

Participants rehearsed the lifting task during a brief practice phase. Seven identical spheres were 3D printed and had a diameter of 82.6 mm. Lead pellets were placed within each sphere such that their mass ranged from 100 to 250 g, increasing in increments of 25 g. Foam padding was used to line the inside of each sphere to ensure a normal centre of gravity. Black plastic 3D-printed handles, which were replicas of force transducers, were attached to each sphere so that participants could lift the objects in the same manner as the experimental stimuli.

#### Experimental stimuli

**Fig. 2:**
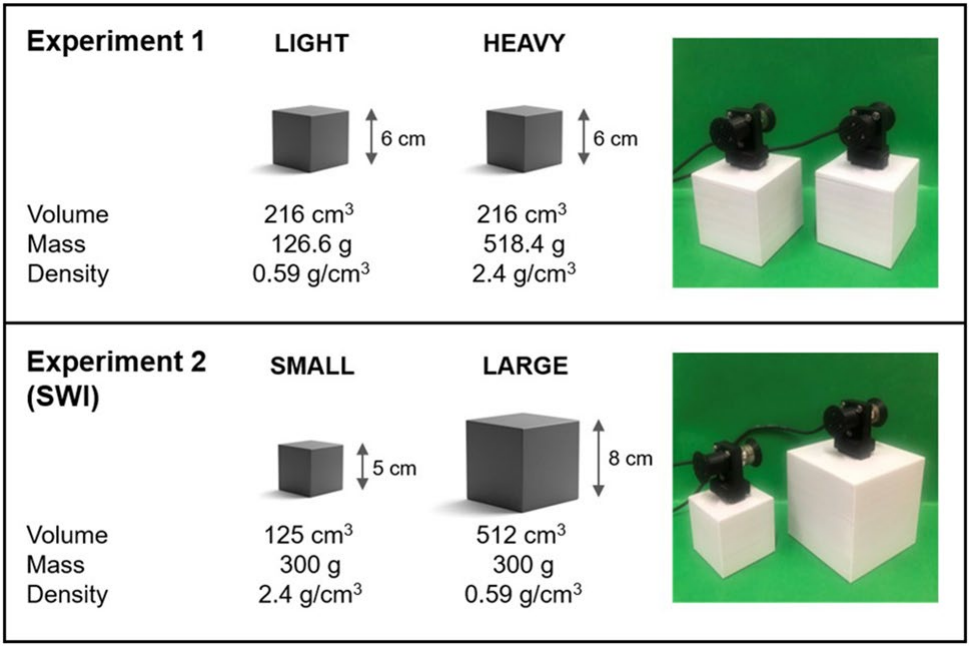
Stimuli used in experiments 1 and 2. Two cubes that were identical in appearance but different in mass were used in experiment 1. Experiment 2 had two cubes of different volumes but had the same mass. The images on the right-hand side of the panel were the stimuli used in the study

In both experiments, we utilised 3D-printed grey cubes (Fig. 2). Experiment 1 had two cubes of the same 6 × 6 × 6 cm size with a volume of 216 cm^3^, but differed in mass, with the light cube weighing 126.6 g and the heavy cube weighing 518.4 g. Experiment 2 had one small cube, measuring 5 × 5 × 5 cm with a volume of 125 cm^3^, and one large cube, measuring 8 × 8 × 8 cm with a volume of 512 cm^3^, both weighing 300 g. The masses reported include the weight of the force transducer handle. Importantly, for both experiments, we also matched the density differences across stimuli, such that the difference in density of the light and heavy cubes (0.59 g/cm^3^ and 2.4 g/cm^3^, respectively) was the same as the difference in density between the large and small cubes (2.4 g/cm^3^ and 0.59 g/cm^3^, respectively), as density can impact perceived weight (Saccone et al. 2019b; Wolf et al. 2018). Therefore, any differences observed across the experiments cannot be attributed to discrepancies in density among the stimuli. Experimental stimuli were lifted from force transducers (Nano17 F/T; ATI Industrial Automation, Garner, North Carolina, USA), which were attached to the top of the cubes.

### Procedure

Participants first familiarised themselves with the lifting procedure using the practice stimuli which were presented twice in a randomised order before the experiments proper began. Half of the participants completed experiment 1 first and the other half completed experiment 2 first. Each experiment consisted of two blocks of 31 lifts for a total of 62 lifts per experiment. Stimuli were presented in a predetermined, pseudo-random order to ensure that there were 15 trials for each cube and Switch condition. To illustrate, for experiment 1, there were 15 light-after-light trials, 15 light-after-heavy, 15 heavy-after-heavy, and 15 heavy-after-light. The first trial of each block was discarded as it was neither a switch nor non-switch trial, given that it began the sequence. For each experiment, we determined two possible presentation orders for the stimuli. These presentation orders were counterbalanced across participants.

**Fig. 3:**
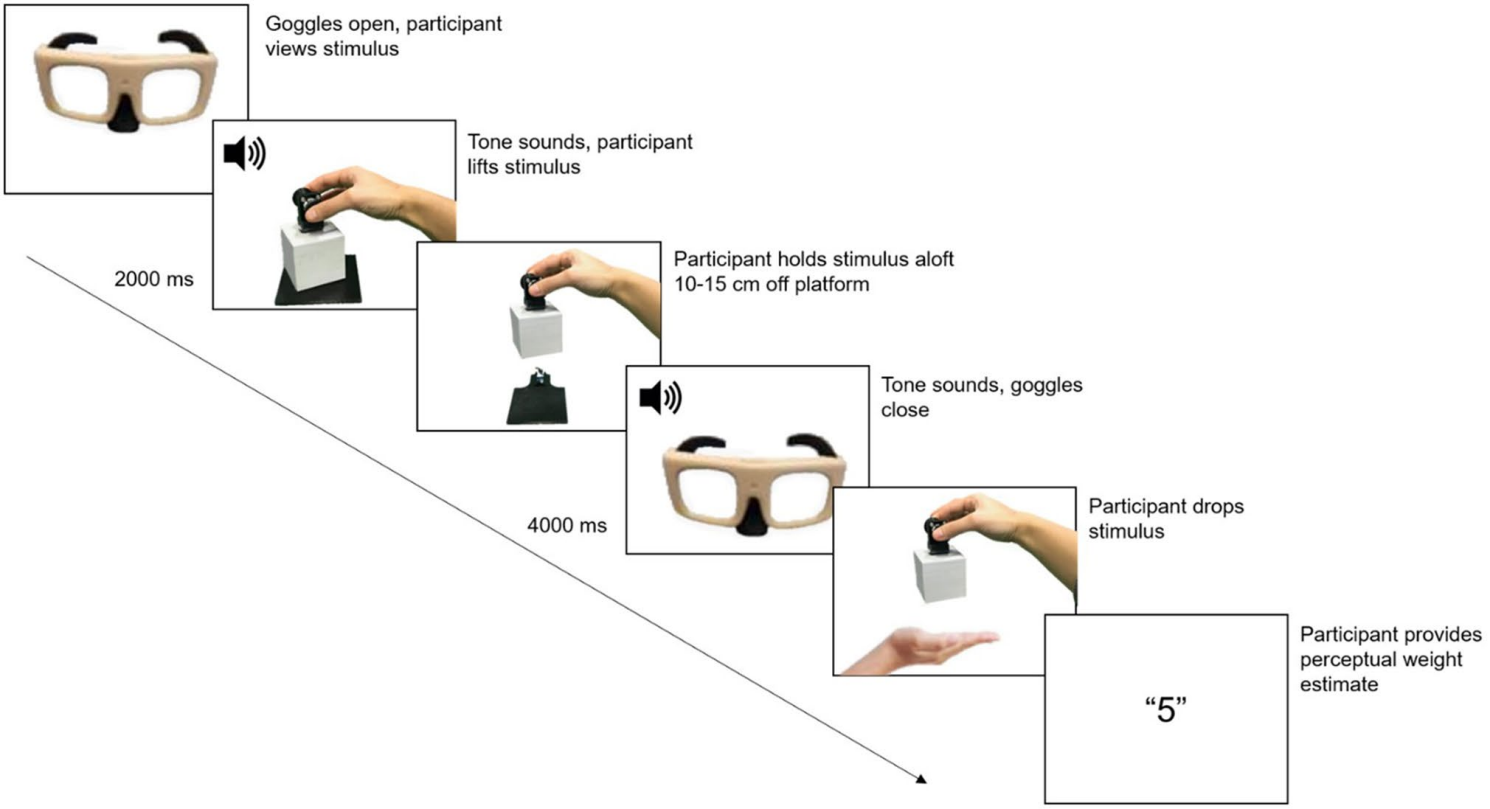
Trial events. Each trial began with the goggles closed. The goggles soon opened and participants passively viewed the stimulus for 2000 ms. An auditory tone then sounded, signalling participants to grasp the force transducer and lift the stimulus vertically. Participants raised and held the stimulus approximately 10–15 cm above the platform. Another auditory tone sounded 4000 ms after the first tone and the goggles closed, occluding vision. Participants then dropped the stimulus into the experimenter’s hand and provided a perceptual weight estimate

In both the familiarisation task and the experiments, participants followed the same lifting procedure, depicted in Fig. 3. Participants wore PLATO goggles (Translucent Technologies Inc., Toronto, Ontario, Canada). These were kept closed and only opened during a trial to prevent participants from forming expectations of weight by observing the experimenter's interactions with the stimuli. For example, the participants were unable to note how easy or difficult it was for the experimenter to replace the stimuli between trials. At the beginning of each trial, participants placed their right finger on the starting position—consisting of a tactile marker positioned 10 cm away from the stimulus. The goggles then opened and participants viewed the stimulus for 2000 ms. Next, an auditory tone sounded, prompting participants to lift the stimulus vertically by grasping the force transducer handle between the thumb and index finger. Participants held the stimulus approximately 10–15 cm above the platform until another auditory tone sounded 4000 ms later. At this point, the goggles closed and participants dropped the stimulus into the experimenter's hand.

Following each lift, perceptual weight estimates were given using absolute magnitude estimates (Zwislocki and Goodman 1980). Namely, participants gave a numerical estimation of the apparent weight of the object where greater numbers represented a heavier weight. Participants were given no restrictions on the numerical scale for their magnitude estimates, allowing them to choose a scale they felt would be most comfortable and precise with, but were instructed to maintain consistency with their selected scale throughout the experiments. It was emphasised that participants should report the weight they were experiencing rather than what they expected or thought was correct. Participants then returned their finger to the starting position in preparation for the next trial.

### Data preperation and analysis

To standardise the participants' perceptual magnitude estimates to the same scale as the other participants, we transformed them into *z*-scores. This was done by subtracting the mean of each participant's total estimate from each individual score and then dividing the result by the standard deviation of all estimates. This was done separately for each experiment.

Force transducer outputs were recorded in MATLAB (The Mathworks, Inc., Natick, Massachusetts, USA) at a rate of 400Hz along the X, Y, and Z axes and were smoothed using a fourth-order, zero-phase lag, low-pass Butterworth filter, with a cut-off frequency of 14 Hz prior to analysis (Flanagan and Beltzner 2000; Saccone et al. 2019a). The force data produced measures of grip force (defined as the force applied normal to the handle’s surface) and load force (defined as the resultant vector of force tangential to the surface of the handle). Force outputs were recorded for the index finger only and were accordingly assumed to be half of what was exerted by both this finger and the thumb.

For each trial, we calculated the peak grip and load forces and the peak rates at which they were applied. To calculate peak grip and load forces, we first plotted the grip and load signals from each lifting trial over time in seconds. The first peaks in these signals were visually identified and selected on the plots by the first rater (R.C.) and the value of each true maximum near the selected peak was recorded using an in-house MATLAB script, reducing human error and increasing accuracy. To establish the peak grip force rate and peak load force rate, each force signal was differentiated with a three-point central difference equation. This process determined the rate applied at each time point in Newtons/seconds (Chouinard et al. 2009; Flanagan and Beltzner 2000; Saccone et al. 2019a), which was again plotted for each trial to record the peak value. To provide an index of reliability for the force data processing method, a second rater (E.S.) independently selected the peaks for all trials from five participants chosen at random. The inter-rater reliability between the two raters was excellent (peak grip force: *r*(600) = 0.95, *p* < 0.001; peak load force: *r*(600) > 0.99, *p* < 0.001, peak grip force rate: *r*(600) > 0.99, *p* < 0.001, peak load force rate: *r*(600) > 0.99, *p* < 0.001).

For each experiment, we carried out a PCA on the four force measures using Varimax rotation in SPSS (Statistical Package for the Social Sciences; Chicago, IL, USA) to determine if they would load onto a single component. A Kaiser–Meyer–Olkin (KMO) measure was calculated to determine the appropriateness of sampling (Kaiser 1974). Components with an eigenvalue above 1 were retained and reported in the factor solution. In both experiments, the PCA yielded a one factor solution using this criterion. A standardised regression score calculated in SPSS was taken as the aggregate force measure used in further statistical analyses (for details on how SPSS calculates this measure, see DiStefano et al. (2009). Nonetheless, in the interest of transparency and allowing one to make comparisons with other studies, we also describe and analyse the four individual force variables separately. However, to combat the issue of multiple statistical tests on the same data, we base our interpretations and conclusions solely on the results of the aggregate force measures.

To quantify the trajectory of possible motor adaptation, we divided the lifting trials into 5 successive bins of 3 trials and calculated the mean values for each bin. For each experiment, these binned means were entered in a 2 (Cube: light vs. heavy (for experiment 1) or small vs. large (for experiment 2)) × 2 (Switch: switch vs. no-switch) × 5 (Bin: 1 vs. 2 vs. 3 vs. 4 vs. 5) repeated-measures ANOVA. Pairwise comparisons were performed with a family-wise Bonferroni correction applied. Greenhouse–Geisser corrections were applied whenever sphericity could not be assumed as determined by a Mauchly’s test.

In addition to the ANOVA, we also performed two correlation-based analyses to investigate whether the aggregate forces applied to a particular mass related to its perceived weight. For the first, we correlated everybody’s binned data in one correlation. This addressed whether there was a general relationship between the two variables. In experiment 1, we did this separately for the light and heavy cubes, given they had different masses. However, these correlations could be influenced by participant-specific effects. To remove these effects, we performed a second analysis to test whether participants, as a group, demonstrated a relationship between the two variables. This was accomplished by first correlating each participant’s binned data to obtain an individual correlation coefficient 2 and then performing a one-sample t-test comparing all participants’ correlation coefficients against zero—effectively treating participants as a random variable. Note all correlations were based on binned rather than trial values so that our correlation-based analyses can be compared with the ANOVA.

Last, we characterised when the four non-aggregated force measures occurred relative to object lift-off. Unfortunately, the photosensing equipment that we had built for recording lift-off times was malfunctioning and deemed unreliable. Therefore, for the purposes of this analysis, we assumed that lift-off occurred when the load force first reached the necessary force to overcome gravity on the stimulus. For experiment 1, this was defined as the time point when the load force signal first reached 0.63 Newtons (i.e., 1.27/2 Newtons) and 2.59 Newtons (i.e., 5.18/2 Newtons) for the light and heavy objects, respectively. For experiment 2, this was defined as the time point when the load force signal first reached 1.5 Newtons (i.e., 3.0/2 Newtons) for the small and large objects. For each participant, we aligned and cropped the grip and load force signals and the grip and load force velocity signals for all trials to align 0.5 s before and 2.0 s after these time points. Following this alignment, we created average signals for each Cube × Switch combination and calculated by automation the time point relative to lift-off when the peak grip and load force and peak rates of these forces occurred. We report the descriptive statistics comprising the mean, standard deviation, and range across participants.

## Results

### Experiment 1: light and heavy cubes

#### Overall summary

The PCA revealed a single component structure in the force data. Switch effects were present in the perceptual weight estimates as well as in the aggregate measure of lifting force. For the light cube, participants exerted greater lifting force and lower perceptual weight estimates in switch compared to no-switch trials. For the heavy cube, participants exerted lower lifting force and higher perceptual weight estimates for the switch compared to no-switch trials. Individual grip and load force variables produced results consistent with the aggregate force measure with the exception of peak load force. Correlation-based evidence for a relationship between applied forces and weight perception was inconsistent. Some evidence was only present for the light cube.

#### Perceptual weight estimates

**Fig. 4:**
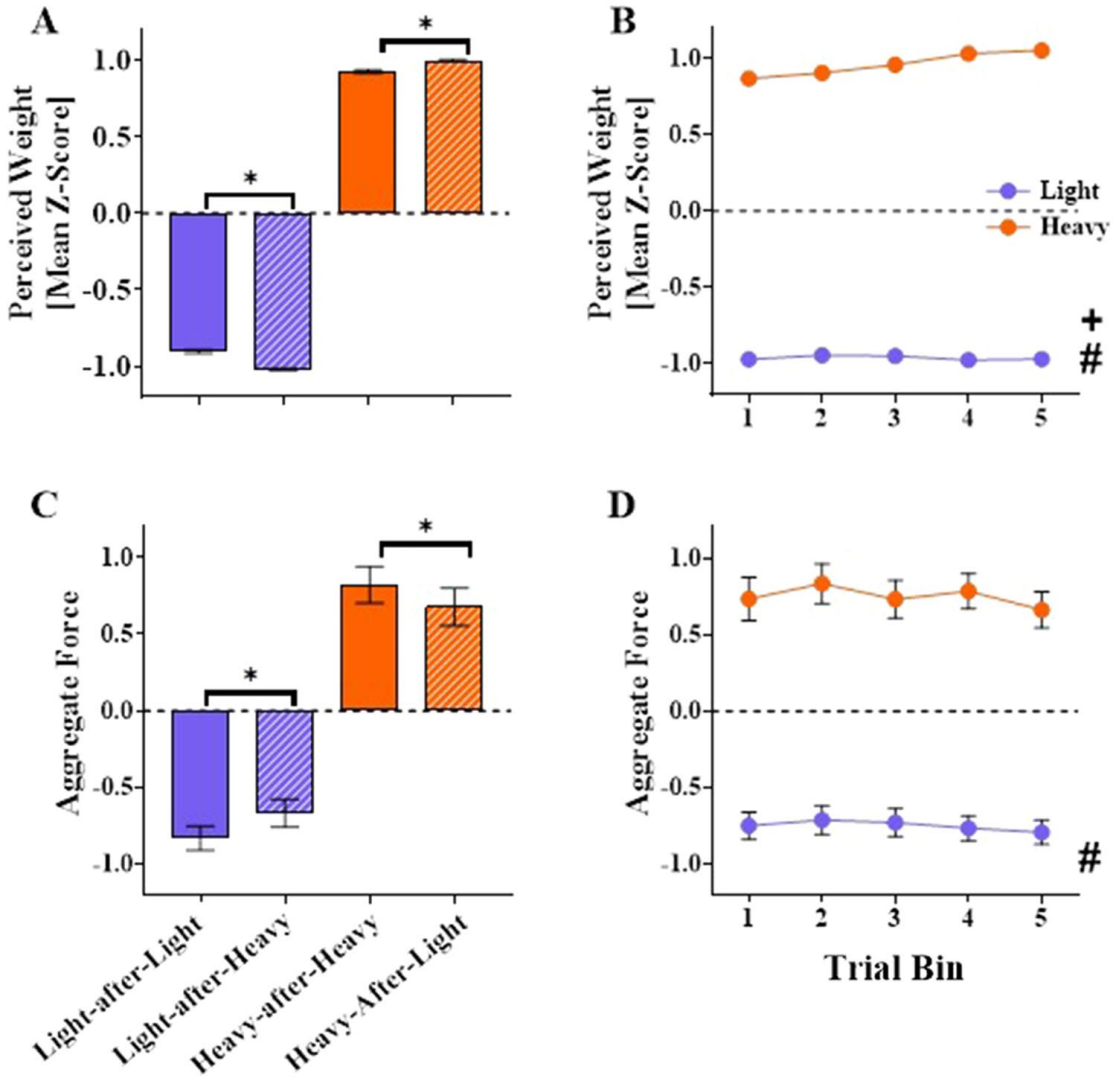
Aggregate forces and perceived weight estimates in experiment 1. Mean standardised perceptual weight estimates (*z*-scores; panels **A**, **B**) and aggregate force variable (standardised regression scores; panels **C**, **D**) produced from the PCA for the light (denoted with purple) and heavy (denoted with orange) cubes in experiment 1. Graphs A and C display Cube × Switch means, i.e., means for no-switch trials for the light cube (light-after-light; purple bars) and heavy cube (heavy-after-heavy; orange bars) and switch trials for the light cube (light-after-heavy; purple striped bars) and heavy cube (heavy-after-light; orange striped bars). Asterisks (*) denote significant pairwise comparisons when examining Cube × Switch interactions (*p* < 0.05, Bonferroni family-wise corrected). Graphs **B** and **D** display the means for light (purple) and heavy (orange) cubes across the five trial bins (averaged across switch conditions). Pound (#) denotes a significant main effect of Cube (*p* < 0.05). Cross (+) denotes a significant main effect of Bin (*p* < 0.05). Dashed grey lines denote the overall mean standardised scores of 0. All error bars denote standard errors around the means

Mean standardised perceptual estimates are displayed in Fig. 4A and B. ANOVA revealed a main effect of Cube, F(1, 33) = 616.55, *p* < 0.001, *η*_*p*_^*2*^ = 0.95, demonstrating greater weight estimates for the heavy cube. There was also a main effect of Switch, F(1, 33) = 5.78, *p* = 0.022, *η*_*p*_^*2*^ = 0.15, with lower perceived weight for switch compared to no-switch trials. There was also a main effect of Bin, F(2, 79) = 4.84, *p* = 0.007, *η*_*p*_^*2*^ = 0.13, with lower estimates for Bin 1 compared to Bin 5 (*p* = 0.045), indicating heavier estimates at the end versus the start of the experiment. None of the other pairwise comparisons were significant (all *p*s ≥ 0.111). There was a significant Cube x Switch interaction, F(1, 33) = 31.58, *p* < 0.001, *η*_*p*_^*2*^ = 0.49. Namely, the light cube was perceived as lighter during switch (light-after-heavy) compared to no-switch (light-after-light) trials (*p* < 0.001, *d* = 1.90) and the heavy cube was perceived as heavier on switch (heavy-after-light) than no-switch (heavy-after-heavy) trials (*p* < 0.001, *d* = 1.17). There was also a Cube x Bin interaction, F(2, 60) = 5.78, *p* = 0.007, *η*_*p*_^*2*^ = 0.15. For the light cube, there were no differences between bins, indicating no change in perceived heaviness over time (all *p*s > 0.99). For the heavy cube, there was a pattern for weight estimates to increase over the course of the experiment. Estimates during Bin 1 were lower than Bins 4 (*p* = 0.03) and 5 (*p* = 0.01) and likewise estimates for Bin 2 were also lower than Bins 4 (*p* = 0.04) and 5 (*p* = 0.01). There were no other significant pairwise comparisons (all *p*s ≥ 0.25) or interactions (both *p*s ≥ 0.30).

#### PCA on the force data

The PCA revealed a single component solution that accounted for 77.4% of the variance of the force data. The KMO value was 0.48. Standardised regression scores were computed to represent a single measure of force for each participant and condition. Figure 4C and D display mean scores from this aggregate measure. ANOVA demonstrated a main effect of Cube, *F*(1, 33) = 616.90, *p* < 0.001, *η*_*p*_^*2*^ = 0.95, reflecting greater force applied for the heavy than the light cube. There was no main effect of Switch, *F*(1, 33) = 0.24, *p* = 0.63, *η*_*p*_^*2*^ = 0.01, or Bin, *F*(3, 93) = 2.25, *p* = 0.09, *η*_*p*_^*2*^ = 0.06. There was a significant Cube x Switch interaction, *F*(1, 33) = 31.80, *p* < 0.001, *η*_*p*_^*2*^ = 0.49. For the light cube, participants applied greater force during switch (light-after-heavy) compared to no-switch (light-after-light) trials (*p* < 0.001, *d* = 0.25). For the heavy cube, lower forces were applied during switch (heavy-after-light) compared to no-switch (heavy-after-heavy) trials (*p* < 0.001, *d* = -0.22). There were no other significant interactions (all *p*s ≥ 0.22).

#### Correlation-based analyses between aggregate forces and perceptual weight estimates

**Fig. 5:**
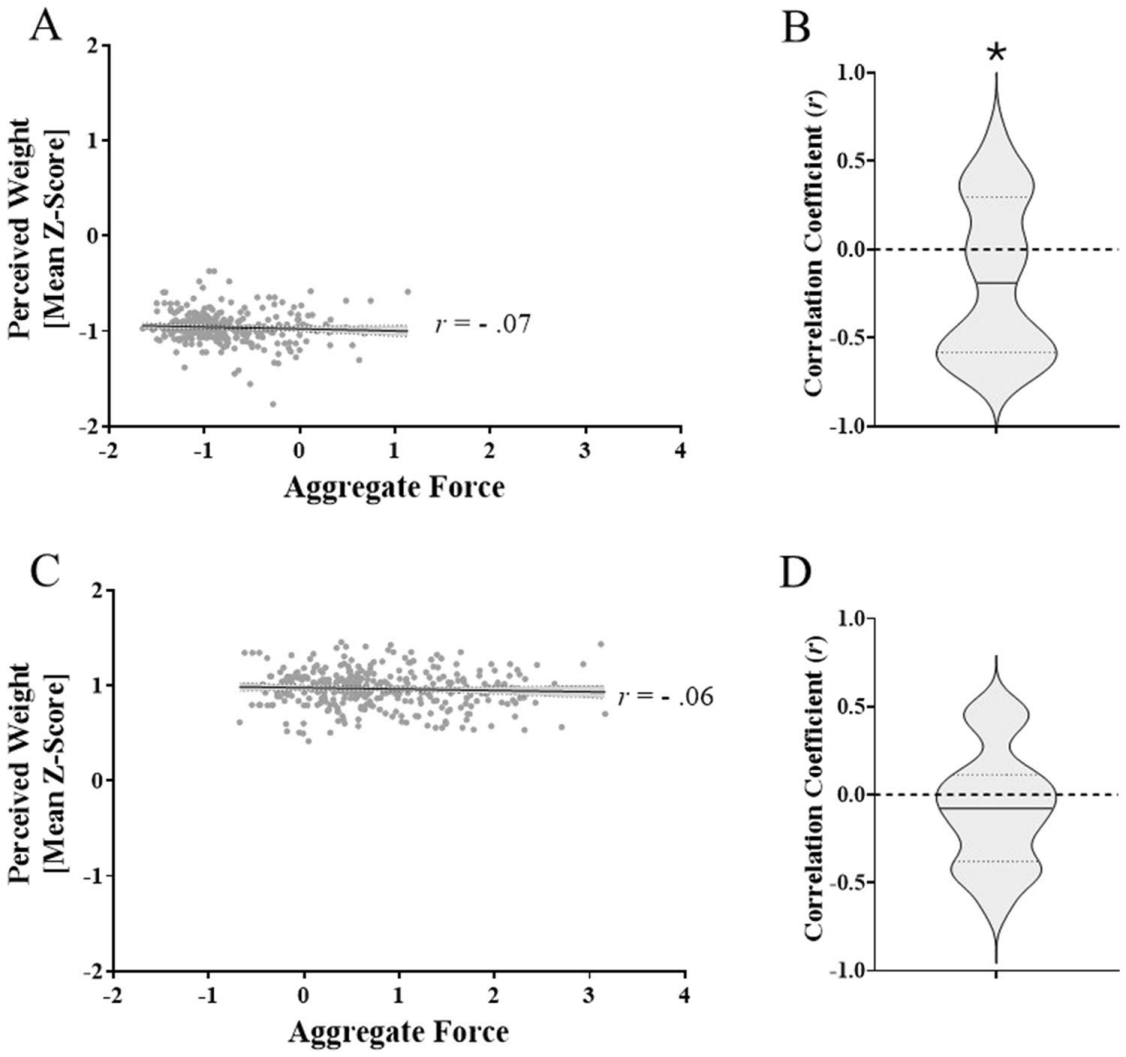
Aggregate forces correlated with perceived weight estimates in experiment 1. Panels ‘**A**’ and ‘**B**’ represent the light cube while ‘**C**’ and ‘**D**’ represent the heavy one. For the violin plots, the solid line represents the mean, while the two dotted lines represent the first and third quartiles. The asterisk (*) denotes a significant effect (*p* < 0.05)

**Fig. 6:**
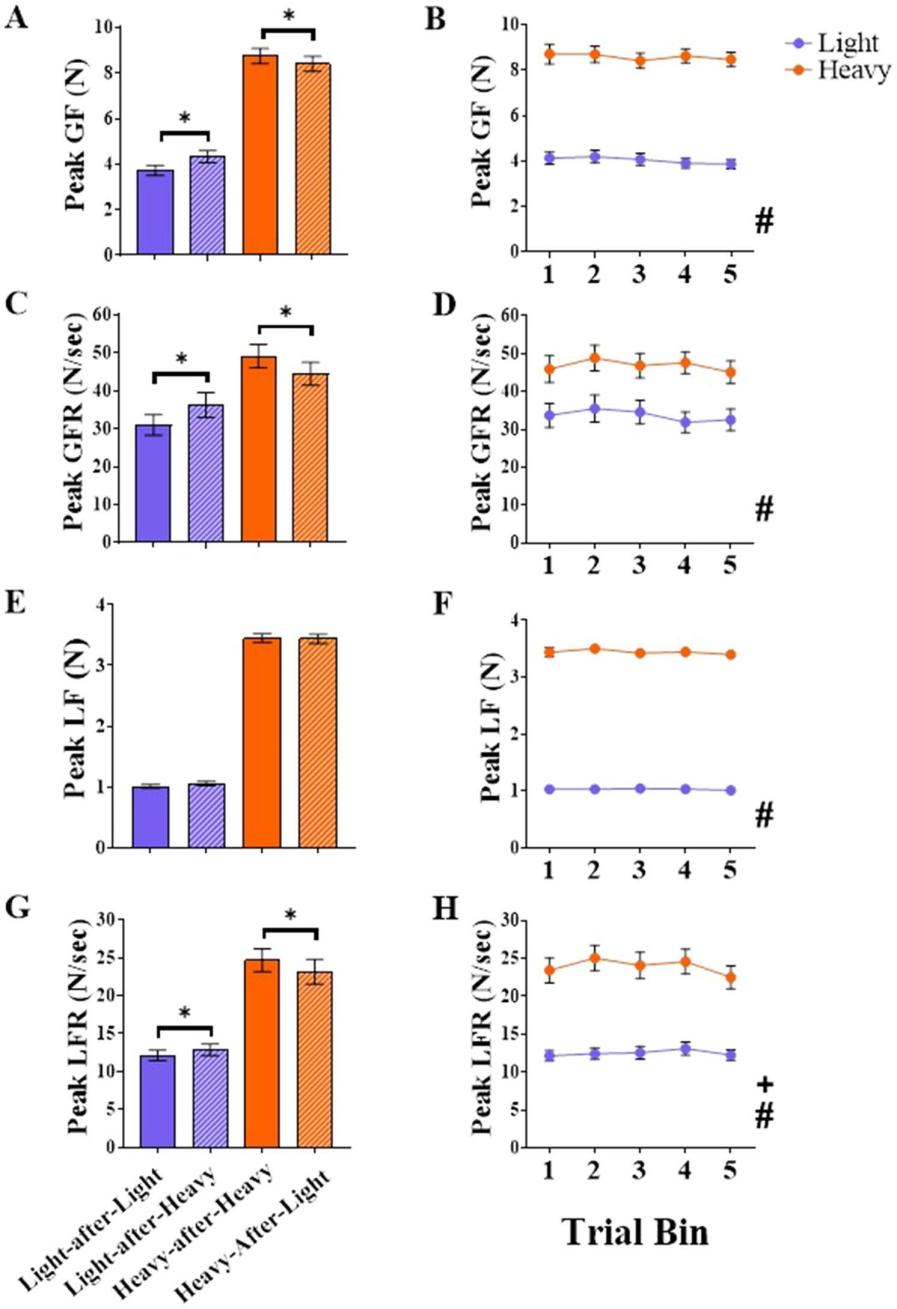
Non-aggregated force measures in experiment 1. Mean peak grip force (GF; in Newtons; panels **A**, **B**), peak grip force rates (GFR; in Newtons/second; panels **C**, **D**), peak load force (LF; in Newtons; panels **E**, **F**), peak load force rates (LFR; in Newtons/second; panels **G, H**) for the light (denoted with purple) and heavy (denoted with orange) cubes in experiment 1. Graphs in the left column (**A, C, E, G**) display Cube × Switch means, i.e., means for no-switch trials for the light cube (light-after-light; purple bars) and heavy cube (heavy-after-heavy; orange bars) and switch trials for the light cube (light-after-heavy; purple striped bars) and heavy cube (heavy-after-light; orange striped bars). Asterisks (*) denote significant pairwise comparisons for examining Cube × Switch interactions (*p* < 0.05, Bonferroni family-wise corrected). Graphs in the right column (**B**, **D, F, H**) show means for light (purple) and heavy (orange) cubes across the five trial bins (averaged across switch conditions). Pound (#) denotes a significant main effect of Cube (*p* < 0.05). Cross (+) denotes a significant main effect of Bin (*p* < 0.05). All error bars denote standard errors around the means

Our first analysis correlating everybody’s binned force aggregates with their binned perceptual weight estimates did not yield significant correlations for either the light (*r*(338) =− 0.07, 95% CI [0.04 to − 0.18], *p* = 0.19; Fig. 5A) or heavy (*r*(338) =− 0.06, 95% CI [0.05 to − 0.16], *p* = 0.31; Fig. 5C) cubes. The one-sample *t*-tests in our second analysis revealed that participants, as a group, demonstrated negative correlations between the forces they applied on the stimulus and how heavy it felt for the light (*t*(33) = − 2.14, *p* = 0.04, *d* = 0.18; Fig. 5B) but not the heavy (*t*(33) = − 1.40, *p* = 0.17, *d* = − 0.24; Fig. 5D) cube. Results for the grip and load peak forces and their peak rates will be reported next. Means for these individual measures are shown in Fig. 6.

#### Peak grip force

There was a main effect of Cube, *F*(1, 33) = 428.47, *p* < 0.001, *η*_*p*_^*2*^ = 0.93, reflecting greater peak grip force applied for the heavy compared with the light cube. There was also a main effect of Switch, *F*(1, 33) = 6.15, *p* = 0.02, *η*_*p*_^*2*^ = 0.16, with participants applying greater peak grip forces during the switch than the no-switch trials. There was no main effect of Bin, *F*(2, 71) = 1.25, *p* = 0.295, *η*_*p*_^*2*^ = 0.04. There was a significant Cube x Switch interaction, *F*(1, 33) = 35.08, *p* < 0.001, *η*_*p*_^*2*^ = 0.52. For the light cube, participants applied greater peak grip force during the switch (light-after-heavy) compared to the no-switch (light-after-light) trials (*p* < 0.001). For the heavy cube, lower peak grip forces were applied during the switch (heavy-after-light) compared to the no-switch (heavy-after-heavy) trials (*p* = 0.001). There were no other significant interactions (all *p*s ≥ 0.07).

#### Peak grip force rates

There was a main effect of Cube, *F*(1, 33) = 80.13, *p* < 0.001, *η*_*p*_^*2*^ = 0.71, with greater rates of grip force applied for the heavy cube. There was no main effect of Switch, *F*(1, 33) = 0.28, *p* = 0.60, *η*_*p*_^*2*^ = 0.01, or Bin, *F*(3, 94) = 1.89, *p* = 0.14, *η*_*p*_^*2*^ = 0.05. There was a significant Cube x Switch interaction, *F*(1, 33) = 33.90, *p* < 0.001, *η*_*p*_^*2*^ = 0.51. For the light cube, grip force rates were higher for the switch (light-after-heavy) than the no-switch (light-after-light) trials (*p* < 0.001). For the heavy cube, rates were lower for the switch (heavy-after-light) compared to the no-switch (heavy-after-heavy) trials (*p* = 0.001). None of the other interactions were significant (all *p*s ≥ 0.34).

#### Peak load force

There was a main effect of Cube, *F*(1, 33) = 2,244, *p* < 0.001, *η*_*p*_^*2*^ = 0.99, reflecting greater peak load forces applied for the heavy than the light cube. There was no main effect of Switch, *F*(1, 33) = 1.60, *p* = 0.214, *η*_*p*_^*2*^ = 0.05, or Bin, *F*(3, 103) = 1.39, *p* = 0.25, *η*_*p*_^*2*^ = 0.04. There were no significant interactions (all *p*s ≥ 0.12).

#### Peak load force rates

There was a main effect of Cube, *F*(1, 33) = 130.93, *p* < 0.001, *η*_*p*_^*2*^ = 0.80, with greater rates of load force applied for the heavy cube. There was no main effect of Switch, *F*(1, 33) = 1.91, *p* = 0.18, *η*_*p*_^*2*^ = 0.06. Although there was a main effect of Bin, *F*(3, 108) = 3.23, *p* = 0.02, *η*_*p*_^*2*^ = 0.09, none of the pairwise comparisons for examining this main effect survived Bonferroni correction (all *p*s ≥ 0.14). There was a significant Cube x Switch interaction,* F*(1, 33) = 12.74, *p* = 0.001, *η*_*p*_^*2*^ = 0.28. For the light cube, the rates of force were higher for the switch (light-after-heavy) compared to the no-switch (light-after-light) trials (*p* = 0.026). For the heavy cube, the rates of force were lower for the switch (heavy-after-light) than the no-switch (heavy-after-heavy) trials (*p* = 0.01). None of the other interactions were significant (all *p*s ≥ 0.13).

#### Temporal characterisation of the four non-aggregated force measures

**Fig. 7:**
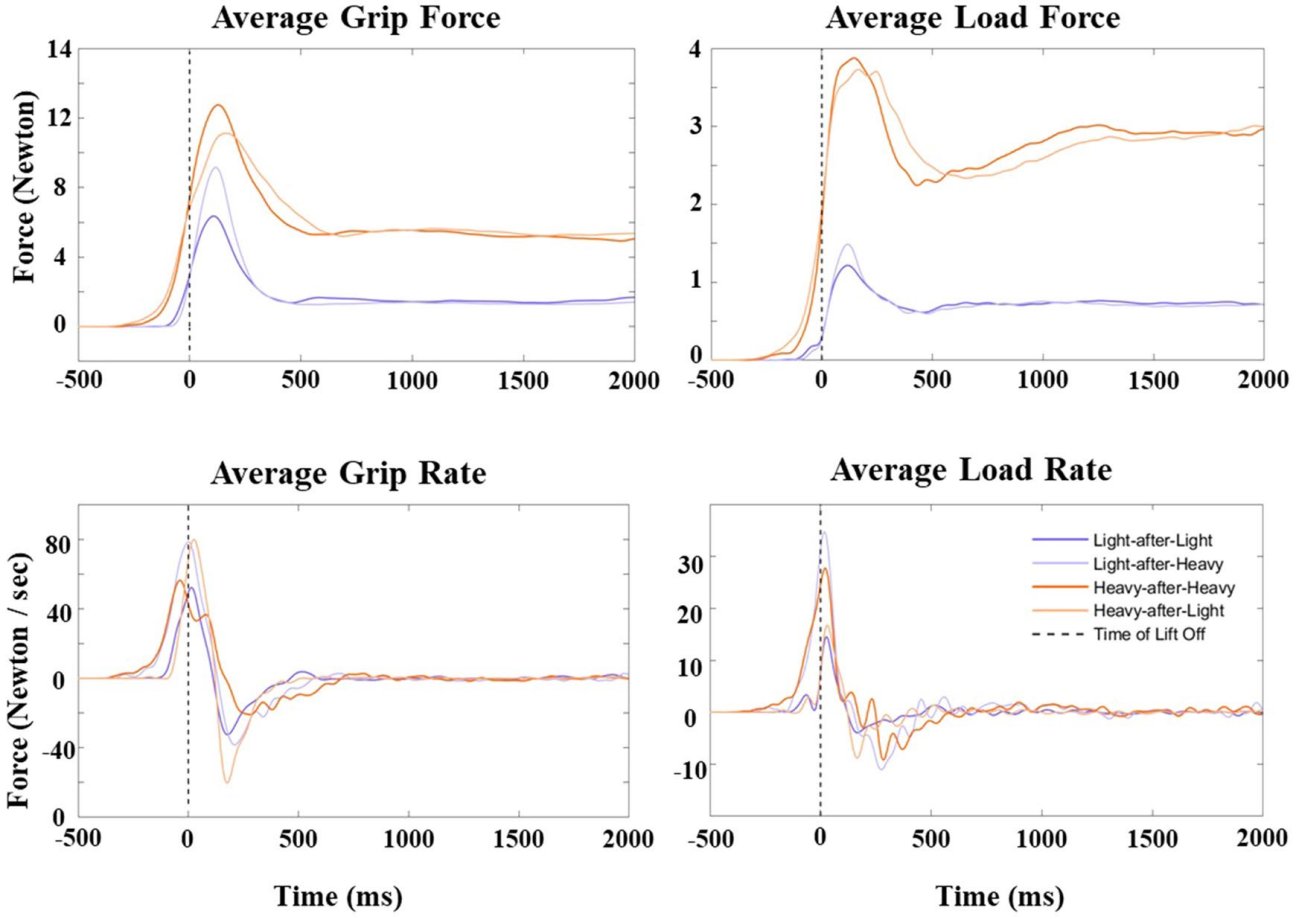
Temporal characterisation of the non-aggregated force measures in experiment 1. The graphs illustrate the average grip force and load force (in Newtons) and the average grip rate and load rate (in Newtons/s) signal traces in a representative participant in experiment 1. The dashed line represents the time of lift-off, as calculated when the applied load force to the object first equalled its mass

The average time when the peak grip force occurred relative to take-off was + 142 ms (*SD* = 52 ms, range: + 73 to + 403 ms). The average time when the peak load force occurred relative to take-off was + 138 ms (*SD* = 75 ms, range: + 35 to + 390 ms). The average time when the peak grip rate occurred relative to take-off was − 35 ms (*SD* = 50 ms, range: − 205 to + 95 ms). The average time when the peak load rate occurred relative to take-off was − 16 ms (*SD* = 31 ms, range: − 138 to + 45 ms). Table 1 provides similar descriptive statistics for each of the different Cube × Switch conditions. Figure 7 illustrates the average signals we plotted for this analysis in a representative participant.

**Table 1:** Descriptive statistics for the temporal characterisation of the non-aggregated force measures in experiment 1

	Time of peak grip force (ms)				Time of peak load force (ms)			
	LL	HH	LH	HL	LL	HH	LH	HL
*Mean*	142	147	153	126	163	120	122	147
*Min*	75	85	93	73	43	73	38	35
*Max*	340	403	215	355	390	258	303	333
*SD*	65	52	28	52	100	38	56	87

	Time of peak grip rate (ms)				Time of peak load rate (ms)			
	LL	HH	LH	HL	LL	HH	LH	HL
*Mean*	− 6	− 61	− 74	1	− 1	− 30	− 33	3
*Min*	− 95	− 188	− 205	− 33	− 43	− 138	− 123	− 13
*Max*	95	− 3	− 10	78	38	13	10	45
*SD*	33	42	51	19	17	34	37	12

The LL, HH, LH, and HL labels refer to the light-after-light, heavy-after-heavy, light-after-heavy, and heavy-after-light conditions, respectively. All times are in milliseconds (ms) relative to lift-off. Negative and positive times reflect times before and after lift-off, respectively

### Experiment 2: Small and large (SWI) cubes

#### Overall summary

Participants reported a SWI, experiencing the small cube as heavier than the large one. The PCA again revealed a single factor structure in the force data. The aggregate measure of lifting force demonstrated that participants exerted greater lifting force for the small compared to the large cube. There was no effect of Bin nor did Bin interact with any other factor, demonstrating that the difference in force output between the two cubes remained consistent over the course of the experiment. The non-aggregated force measures tended to yield similar results as the aggregated one. The correlation-based analysis revealed that participants perceived the stimuli as being lighter when they applied more force on them.

#### Perceptual weight estimates

**Fig. 8:**
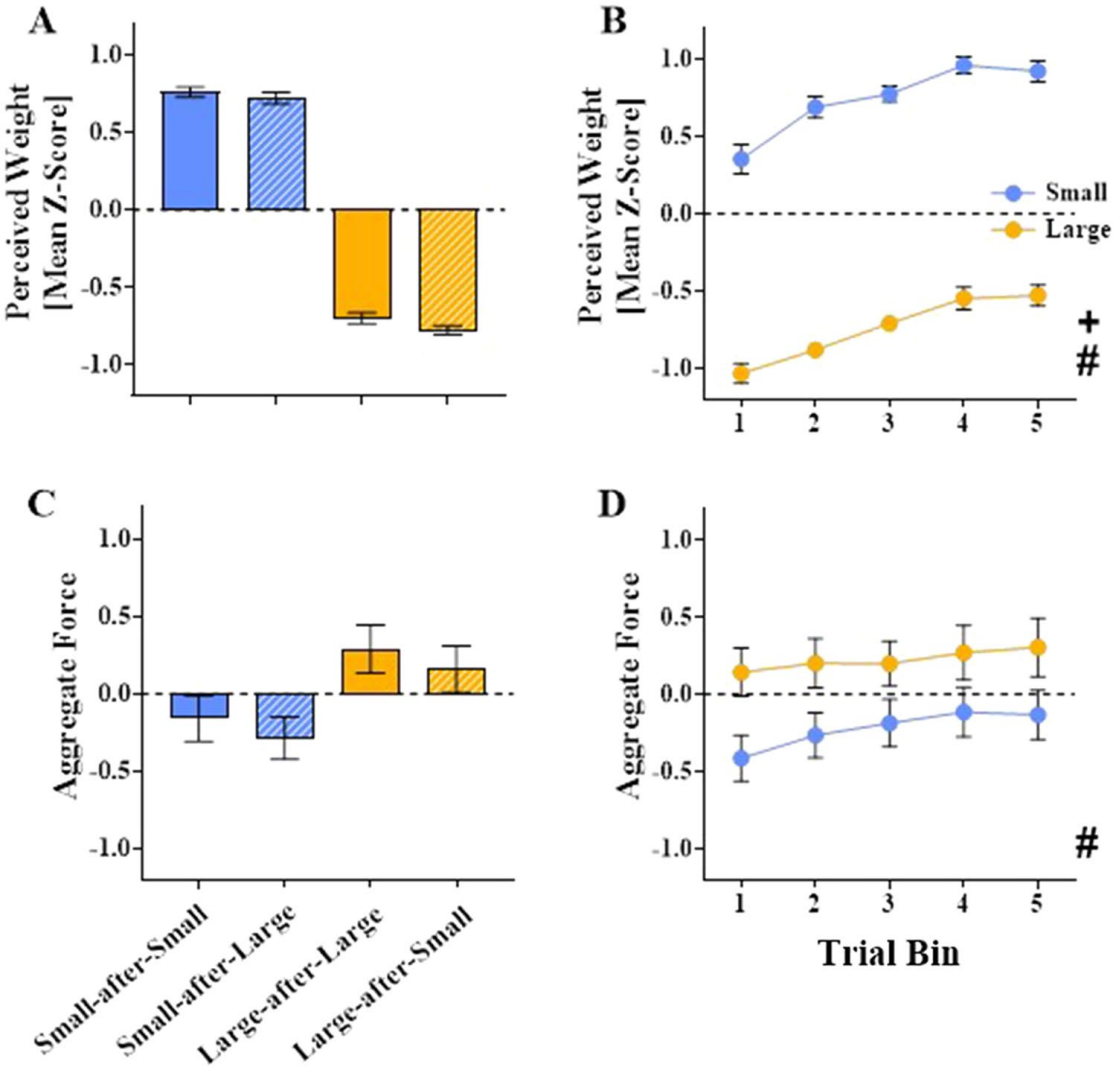
Aggregate forces and perceived weight estimates in experiment 2. Mean standardised perceptual weight estimates (*z*-scores; panels **A**, **B**) and aggregate force variable (standardised regression scores; panels **C**, **D**) produced from the PCA for the small (denoted with blue) and large (denoted with gold) cubes in experiment 2. Graphs **A** and **C** display Cube × Switch means, i.e., means for no-switch trials for the small cube (small-after-small; blue bars) and large cube (large-after-large; gold bars) and switch trials for the small cube (small-after-large; blue striped bars) and large cube (large-after-small; gold striped bars). Graphs **B** and **D** display the means for small (blue) and large (gold) cubes across the five trial bins (averaged across switch conditions). Pounds (#) denote a significant main effect of Cube (*p* < 0.05). A cross (+) denotes a significant main effect of Bin (*p* < 0.05). Dashed grey lines denote the overall mean standardised scores of 0. All error bars denote standard errors around the means

The mean standardised perceptual estimates are displayed in Fig. 8A and B. ANOVA revealed a main effect of Cube, *F*(1, 33) = 813.18, *p* < 0.001, *η*_*p*_^*2*^ = 0.96. Participants reported the small cube as being heavier than the large cube, consistent with the SWI. There was also a main effect of Switch, *F*(1, 33) = 4.78, *p* = 0.036, *η*_*p*_^*2*^ = 0.13, driven by lower perceived weight for switch compared to no-switch trials. There was also a main effect of Bin, *F*(2, 66) = 15.84, *p* < 0.01,* η*_*p*_^*2*^ = 0.32 (Greenhouse–Geisser corrected), reflecting greater estimates as the experiment progressed. Specifically, estimates for Bin 1 were lower than all other bins (all *p*s ≤ 0.005) and Bin 2 was lower than Bins 4 (*p* = 0.004) and 5 (*p* = 0.01; all other pairwise comparisons: *p* ≥ 0.08). There were no significant interactions (all *p*s ≥ 0.35).

#### PCA on the force data

The PCA revealed a single factor solution that accounted for 69.1% of the variance in the force data. The KMO value was 0.57. Once again, the standardised regression scores were computed as an aggregate force variable. The mean scores from this aggregate measure are displayed in Fig. 8C and D. ANOVA demonstrated a main effect of Cube, *F*(1, 33) = 83.97, *p* < 0.001, *η*_*p*_^*2*^ = 0.72, reflecting greater force applied when lifting the large relative to the small cube. There was also a main effect of Switch, *F*(1, 33) = 11.87, *p* = 0.002, *η*_*p*_^*2*^ = 0.27, with less force applied during switch trials. There was no main effect of Bin, *F*(3, 87) = 2.14, *p* = 0.11, *η*_*p*_^*2*^ = 0.06 (Greenhouse–Geisser corrected). There were no significant interactions (all *p*s ≥ 0.37).

#### Correlation-based analyses between aggregate force and perceptial weight estimates

**Fig. 9:**
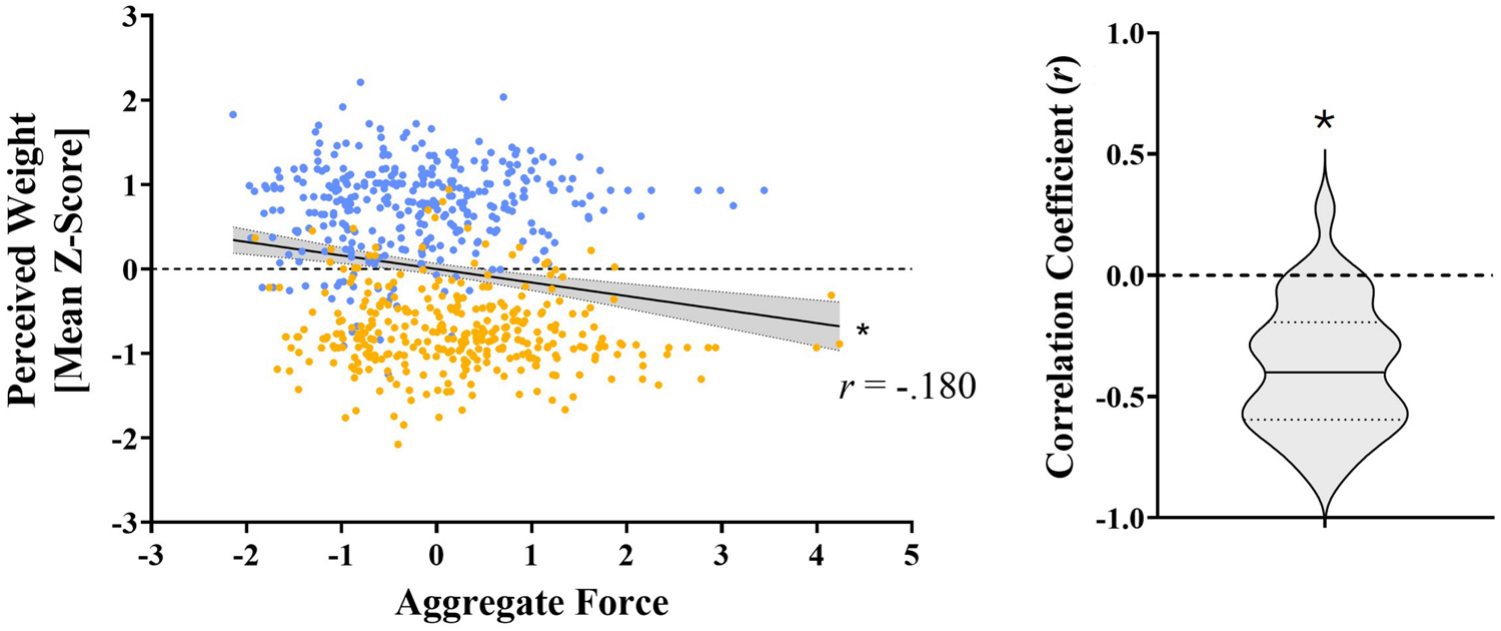
Aggregate forces correlated with perceived weight estimates in experiment 2. The panel on the left (**A**) shows a scatterplot of binned perceived weight estimates plotted as a function of the binned aggregated force with a regression line and 95% confidence intervals. Data points in gold represent the large sphere while those in blue represent the small sphere. The panel on the right (**B**) depicts a violin plot of the individual correlation coefficients. For the violin plot, the solid line represents the mean, while the two dotted lines represent the first and third quartiles. Asterisks (*) denote a significant effect (p < 0.05)

**Fig. 10:**
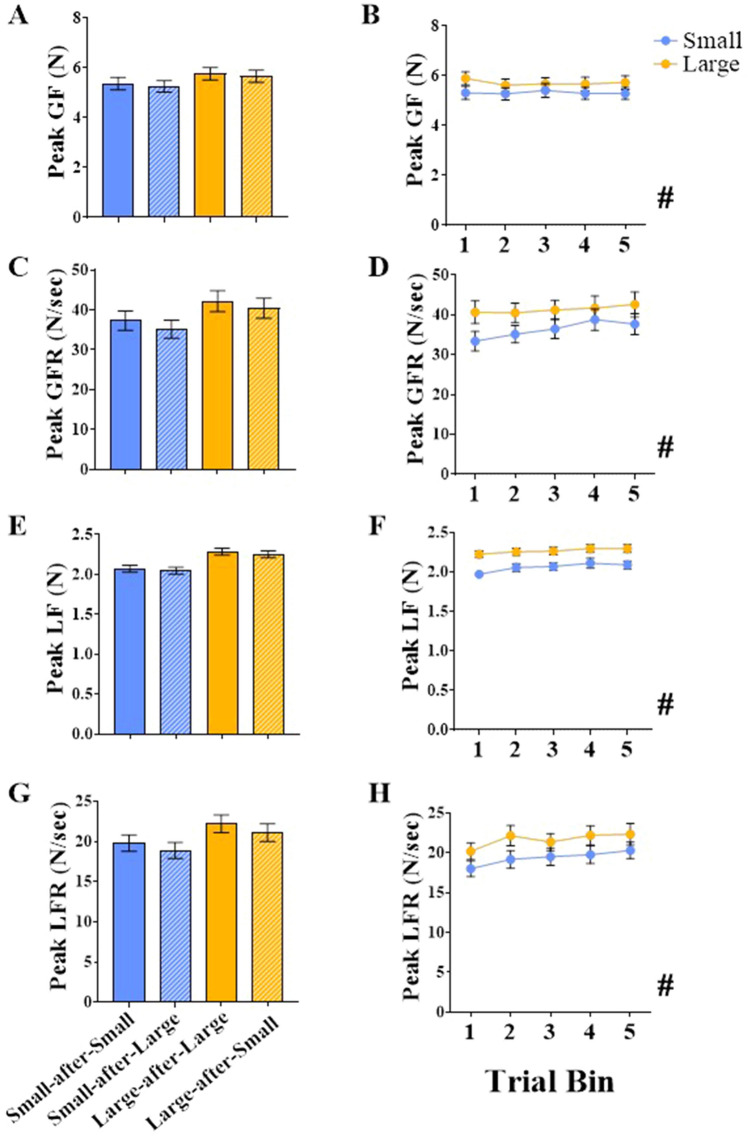
Non-aggregated force measures in experiment 2. Mean peak grip force (GF; in Newtons; panels **A, B**), peak grip force rates (GFR; in Newtons/second; panels **C, D**), peak load force (LF; in Newtons; panels **E, F**), peak load force rates (LFR; in Newtons/second; panels **G, H**) for the small (denoted with blue) and large (denoted with gold) cubes in experiment 2. Graphs in the left column (**A, C, E, G**) display Cube × Switch means, i.e., means for no-switch trials for the small cube (small-after-small; blue bars) and large cube (large-after-large; gold bars) and switch trials for the small cube (small-after-large; blue striped bars) and large cube (large-after-small; gold striped bars). Graphs in the right column (**B, D, F, H**) shown means for the small (blue) and large (gold) cubes across the five trial bins (averaged across switch conditions). Pounds (#) denote a significant main effect of Cube (*p* < 0.05). All error bars denote standard errors around the means

Our first analysis correlating everybody’s binned force aggregates with their binned perceptual weight estimates yielded a negative correlation, *r*(678) = − 0.18, 95% CI [0.25 to 0.11], *p* < 0.001, whereby the stimuli felt lighter as a function of the forces that were applied on them (Fig. 9A). The one-sample t-test in our second analysis revealed that participants, as a group, demonstrated negative correlations between the forces they applied on the stimuli and how heavy they felt (*t*(33) = − 8.37, *p* < 0.001, *d* = − 1.43; Fig. 9B). Results for the grip and load peak forces and their peak rates will be reported next. Means for these individual measures are shown in Fig. 10.

#### Peak grip force

There was a main effect of Cube, *F*(1, 33) = 21.68, *p* < 0.001, *η*_*p*_^*2*^ = 0.40, driven by greater peak grip forces for the large compared to the small cube. There was no main effect of Switch, *F*(1, 33) = 3.38, *p* = 0.08, *η*_*p*_^*2*^ = 0.09, or Bin, *F*(4, 132) = 0.47, *p* = 0.76, *η*_*p*_^*2*^ = 0.01. There were no significant interactions (all *p*s ≥ 0.38).

#### Peak grip force rates

There was a main effect of Cube, *F*(1, 33) = 45.59, *p* < 0.001, *η*_*p*_^*2*^ = 0.58, with greater rates of grip force applied for the large compared to the small cube. There was also a main effect of Switch, *F*(1, 33) = 9.03, *p* = 0.01, *η*_*p*_^*2*^ = 0.22, with lower rates of force applied during switch compared to no-switch trials. There was no main effect of Bin, *F*(3, 87) = 1.90, *p* = 0.14, *η*_*p*_^*2*^ = 0.05. There were no significant interactions (all *p*s ≥ 0.07).

#### Peak load force

There was a main effect of Cube, *F*(1, 33) = 124.41, *p* < 0.001, *η*_*p*_^*2*^ = 0.79, driven by the application of greater forces for the large cube. There was no main effect of Switch, *F*(1, 33) = 4.12, *p* = 0.05, *η*_*p*_^*2*^ = 0.11. Although there was a main effect of Bin, *F*(3, 97) = 3.15, *p* = 0.03, *η*_*p*_^*2*^ = 0.09, none of the pairwise comparisons for examining this main effect survived Bonferroni corrections (all *p*s ≥ 0.14). There were no significant interactions (all *p*s ≥ 0.50).

#### Peak load force rates

There was a main effect of Cube, *F*(1, 33) = 59.34, *p* < 0.001, *η*_*p*_^*2*^ = 0.64, reflecting higher rates of force when lifting the large cube. There was also a main effect of Switch, *F*(1, 33) = 15.52, *p* < 0.001, *η*_*p*_^*2*^ = 0.32, with lower rates of force applied during switch trials. The was also a main effect of Bin, *F*(2, 78) = 4.10, *p* = 0.015, *η*_*p*_^*2*^ = 0.11. None of the pairwise comparisons for examining this main effect survived Bonferroni corrections (all *p*s < 0.54). There were no significant interactions (all *p*s < 0.07).

#### Temporal characterisation of the four non-aggregated force measures

**Fig. 11:**
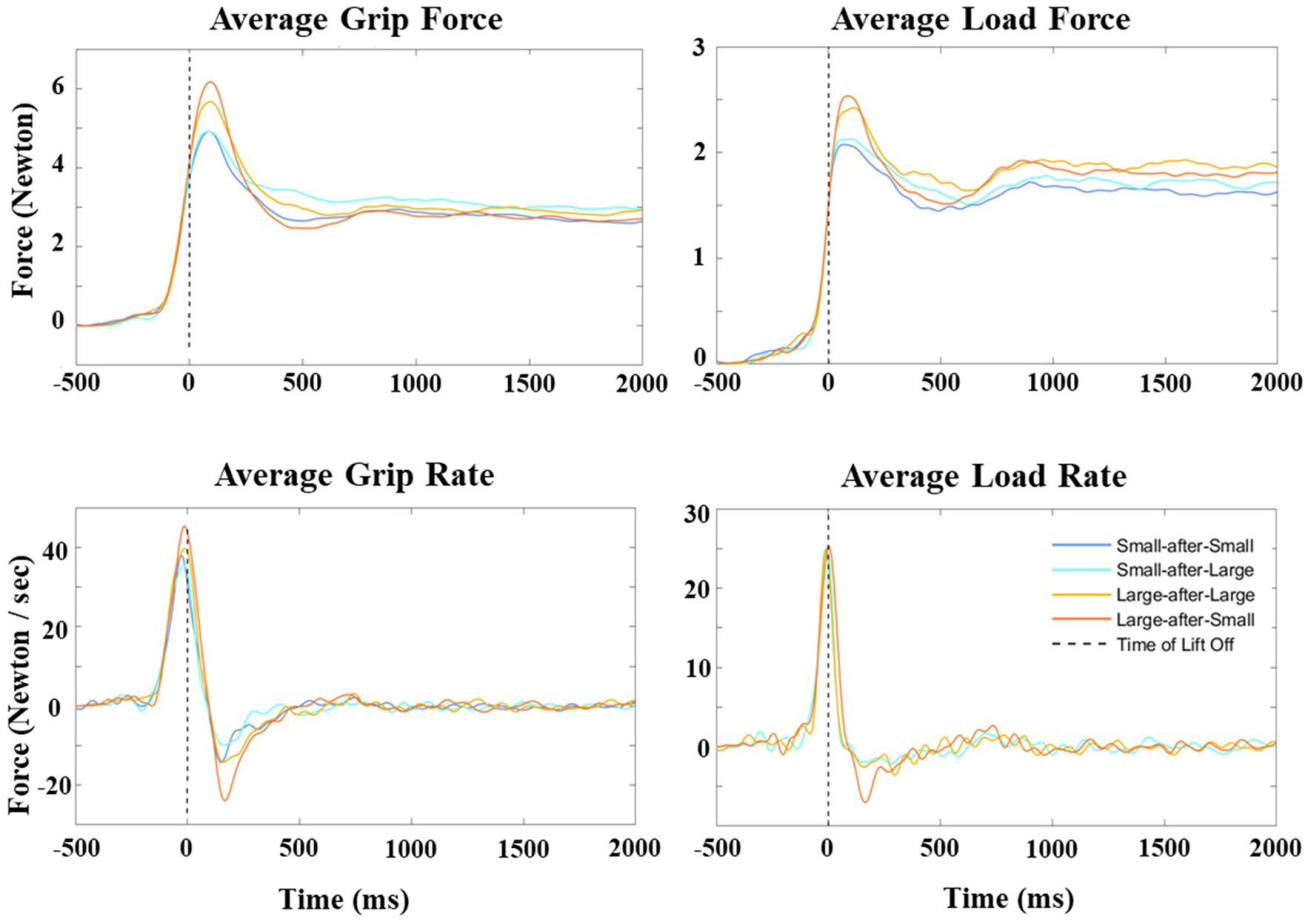
Temporal characterisation of the four force measures. Average grip force and load force (in Newtons), and average grip rate and load rate (in Newtons/s) for a representative participant in experiment 2. The dashed line represents time of lift-off, as calculated when the applied load force to the object first equalled its mass

The average time when the peak grip force occurred relative to take-off was + 112 ms (*SD* = 31 ms, range: + 70 to + 205 ms). The average time when the peak load force occurred relative to take-off was + 107 ms (*SD* = 44 ms, range: + 38 to + 310 ms). The average time when the peak grip rate occurred relative to take-off was − 37 ms (*SD* = 24 ms, range: − 130 to + 10 ms). The average time when the peak load rate occurred relative to take-off was − 16 ms (*SD* = 18 ms, range: − 85 to + 23 ms). Table 2 provides similar descriptive statistics for each of the different Cube x Switch conditions. Figure 11 illustrates the average signals we plotted for this analysis in a representative participant.

## Discussion

This research aimed to re-examine SMT as an explanation for perceiving the weight of objects. Many of the results supported SMT. In the ensuing discussion, we provide an overview of the key findings from the two experiments. We then examine how the results challenge previous research in this area. Next, we consider the role of visual dominance for explaining why motor adaptation was not observed. Following this, we cover the merits of SMT for explaining weight perception and illusions. After, we emphasise the strengths of utilising a force aggregate over individual force measures, discuss non-significant findings and study limitations, and conclude with final remarks.

### Support for SMT

In experiment 1, ANOVA revealed differences in both force application and weight perception in the switch conditions for both the light and heavy cubes, consistent with the predictions of SMT and findings from previous research (Johansson and Westling 1988; van Polanen and Davare 2015). Correlation-based analyses further demonstrated some evidence for a relationship between lifting forces and perceived weight for the light cube, where its perceived weight decreased as lifting forces increased. Similarly, in experiment 2, ANOVA indicated that participants perceived the large cube as lighter than the small one. Contrary to many SWI studies (e.g. Flanagan & Beltzner 2000; Grandy & Westwood 2006), participants did not demonstrate motor adaptation. Instead, they consistently applied more force to the large cube. Additionally, correlation-based analyses highlighted a negative relationship between perceived weight and the forces applied to the cubes.

Another consideration is the timing of when our force measures occurred relative to when weight perception occurred. Establishing if a *cause* occurred before its *effect* is necessary for establishing causality. The precise timing when weight perception first occurred was not measured in this study. However, previous research by Plaisier et al. (2019) suggests that this can occur within 330–400 ms after lift-off. Notably, the peaks of the force measures obtained in our study occurred earlier than this time frame (Figs. 7 and 11; Tables 1 and 2), suggesting a possible causal relationship between applied forces and weight perception. It should be noted that participants provided magnitude estimates of weight later than this time point. Hence, it is always possible that the participants’ magnitude estimates may reflect a perception of weight that differed from the moment they first perceived weight, which presumably gets continuously revised as time goes by according to Dennett’s multiple drafts theory (1991). The use of occluding goggles likely minimised the influence of extraneous variables on the perceptual weight measures between the moment participants first perceived weight and when they reported their weight estimates.

**Table 2:** Descriptive statistics for the temporal characterisation of the non-aggregated force measures in experiment 2

	Time of peak grip force (ms)				Time of peak load force (ms)			
	LL	SS	LS	SL	LL	SS	LS	SL
*Mean*	118	109	110	117	108	99	112	107
*Min*	73	70	73	70	50	40	38	55
*Max*	205	178	200	233	310	250	280	253
*SD*	36	26	29	41	43	40	54	36

	Time of peak grip rate (ms)				Time of peak load rate (ms)			
	LL	SS	LS	SL	LL	SS	LS	SL
*Mean*	− 33	− 41	− 37	− 31	− 13	− 19	− 17	− 12
*Min*	− 115	− 120	− 130	− 145	− 75	− 55	− 85	− 68
*Max*	− 3	10	3	0	15	23	13	20
*SD*	23	25	25	29	17	19	18	17

The LL, SS, LS, and SM labels refer to the large-after-large, small-after-small, large-after-small, and small-after-large conditions, respectively. All times are in milliseconds (ms) relative to lift-off. Negative and positive times reflect times before and after lift-off, respectively

In summary, ANOVA indicated differences in force application and weight perception that are consistent with SMT predictions. Correlational-based analyses provide some support for a relationship between the two variables. A temporal analysis on the force measures suggests a causal relationship between applied forces and weight perception—assuming weight perception first occurred within 330–400 ms after lift-off.

### Contradictions with previous research

Our findings challenge the notion of a dissociation between perceptual and sensorimotor predictions of weight as proposed by Flanagan and Beltzner (2000). Contrary to their findings, our aggregate lifting force measure showed no motor adaptation over time. Across all trials, from the first to the last bin, participants consistently applied less force to the small cube and more force to the large one. The lack of motor adaptation cannot be accounted for as merely an artifact arising from aggregating different force measures. The individual force measures—peak load force, peak load force rate, peak grip force, and peak grip force rate—tended not to adapt. The absence of motor adaptation is not unique (Engel et al. 2020; Platkiewicz and Hayward 2014). In particular, a previous SWI study conducted in our laboratory also did not show motor adaptation for any force measure (Saccone et al. 2019a).

One potential explanation for discrepant findings relates to differences in experimental set-ups, particularly the use of inter-trial blinding. In both this study and the Saccone et al. (2019a) study, participants were only able to view the stimulus they were about to lift during a trial, without observing any other stimuli, the experimenter, or the experimenter’s interactions with the stimuli. This consideration is important because previous research demonstrated that the lifting dynamics for an object can be influenced by observing how others interact with it (Buckingham et al. 2014)—which can potentially introduce unintended experimenter effects on how participants perform their lifts and provide perceptual estimates (Firestone & Scholl 2016). We safeguarded against these extraneous influences, as in the Saccone et al. (2019a) study. In both studies, motor adaptation was not observed.

Moreover, we would argue it seems unlikely that human brains would evolve in a way that divorces perceptual experiences of object weight and how people apply forces on them. While Flanagan and Beltzner (2000) suggest an independence of these processes, they do not offer a mechanism for how this independence operates or why our brains would be wired this way. These omissions are significant, especially considering that SMT provides a tangible framework for explaining weight perception.

### A potential role of visual dominance

The absence of motor adaptation prompts the following question: why would there be no motor adaptation? A possible explanation lies in the dominance of vision to override motor adaptation. As highlighted by Stokes and Biggs (2014), vision provides certain spatial information about objects and our environment that cannot be substituted by any other sense, particularly outside of peripersonal space. Touch can provide geometrical information about objects, such as their size, shape, and orientation, but only within peripersonal space using an egocentric frame of reference (i.e., relative to the self). On the other hand, vision can provide the same information both inside and outside peripersonal space using both egocentric and allocentric (i.e., relative to other objects) reference frames.

It then follows that vision could be better placed than touch, and the other senses, to form expectations about the weight of objects around us—enabling us to see an object from a distance, go to it, and apply optimal lifting forces to use it quickly. Undoubtedly, this would have been important to our survival, for example, in effectively finding, lifting, and throwing a weapon, such as a rock, to defend ourselves from an attacker, or to hunt. Thus, it makes more sense to us that the visual properties of objects that inform us about weight would override motor adaptation. In experiment 1, participants kept applying initial forces that reflected associations between weight and the cube that they visually recognised as being the same cube as the last trial. In experiment 2, participants saw the size of the cube that they were to lift and kept applying initial forces that reflected associations between size and weight that is frequently useful in the real world. Indeed, there is ample evidence documenting close ties between vision and how people lift objects. Previous research indicates that vision plays a crucial role in controlling the kinematic aspects of object manipulation (Johansson 2002; Johansson and Westling 1988) and generating sensorimotor predictions for lifting objects (e.g., Buckingham and Goodale 2010a).

### The advantages of using an aggregate measure

Our study also addressed a significant concern raised in the introduction regarding interpretations based on individual force measures.

In experiment 1, we observed a main effect of Cube and a Cube × Switch interaction across all individual force variables, except for peak load force, which exhibited no interaction. A main effect of Switch was evident in peak grip force but not in any other force variable. Moreover, a main effect of Bin was observed in peak load force rates, which was not present in any of the other force measures. In experiment 2, statistical inconsistencies persisted despite all individual force measures showing a main effect of Cube. A main effect of Bin was seen in peak load forces and peak load force rates, but not in peak grip forces or grip force rates. Peak rates for both grip and load forces showed a main effect of Switch, with lower force rates applied for the switch trials for both cubes. However, this behaviour was not observed in peak grip forces and peak load forces.

Such inconsistencies are important to consider. If we had solely reported load force measures, as done previously in many studies (Fig. 1; Table 3 in “Appendix”), experiment 2 would have indicated a main effect of Bin, implying some motor adaptation, and partial evidence for switch effects. However, these findings are contradicted by the aggregate measure. Such inconsistencies underscore the problem of relying on an individual force variable, particularly when the rationale for their selection and exclusion is not explicitly stated. If interpretations are based on an individual measure, then findings may vary depending upon which ones are selected. To address this issue, we propose a solution: the utilisation of an aggregate measure of the four most commonly used force variables, representing a single construct. By adopting this approach, the issue of inconsistent and inadequately justified measures can be addressed, leading to a clearer and more coherent understanding of how forces might relate to weight perception.

### Limitations

It is worth noting that the reported correlations in experiment 1 were not as pronounced as those in experiment 2. We suggest this is because the changes in aggregated forces arising from the unexpected changes in mass in experiment 1 were less in magnitude than those arising from differences in cube size in experiment 2 (for similar findings and explanations, see van Polanen and Davare 2015). To explain, the unexpected changes in mass in experiment 1 caused participants to apply 6.4% more force for the light cube and 5.7% less force for the heavy cube. In contrast, changes in cube size in experiment 2 caused participants to apply 17.2% more force to lift the large compared to the small cube. Consequently, the effects that applied forces could exert on weight perception were smaller in experiment 1. We provide an analysis in the Supplementary Material that further examines this idea. This additional analysis reveals that the experimental design in experiment 2 did indeed influence forces to a greater extent. The difference in power between experiments 1 and 2 underscore the importance of having an experimental manipulation in place that alters lifting forces substantially enough so one could statistically detect how they might correlate with perceived weight with a feasible number of participants.

Moreover, Weber’s law would predict that changes in applied forces from the unexpected changes in mass in experiment 1 and changes in cube size in experiment 2 would be imperceptible and perceptible, respectively (Weber 1831). To explain, Weber suggested that there must be at least a 10% difference in magnitude between two stimuli in order for them to be perceived differently, a.k.a. the point for a just noticeable difference. This implies that participants might need at least a 10% difference in applied forces for these forces to be perceptually noticeable. It could be the case that SMT might operate more strongly when participants are aware of the changes in forces that are applied on objects, as might be the case in experiment 2.

There are two other limitations that are also worth considering. First, while the PCAs were significant, it is worth noting that their KMO values, though still surpassing the recommended threshold (Kaiser 1974), were on the lower end as to what is deemed acceptable. In the future, the collection of greater samples could increase KMO values to establish more confidence in the PCAs. Second, splitting the data in half to account for two different masses in experiment 1 could have also contributed to less powerful correlations in this experiment relative to experiment 2.

### Concluding remarks

Our study provides compelling evidence that weight perception and the SWI are influenced by forces exerted during object lifting, in accordance with SMT predictions. As such, we argue for a re-evaluation of SMT. We further provide reasons as to why discrepancies in motor adaptation exist in the literature and suggest new approaches for future investigations.

### Electronic supplementary material

Below is the link to the electronic supplementary material.

Supplementary file1 (DOCX 16 kb)

## Data Availability

Data is available through the Open Science Framework: https://osf.io/eyr5v.

## Appendix

See Table

**Table 3:** Force measures used in previous research

References	LF	LFP	LFR	LFRP	LF_1st_	LFRP_Diff_	GFP	GFR	GFRP	GF_1st_	GFRP_Diff_	Other
Baugh et al. (2016)	✓			✓	✓							
Buckingham and Goodale (2010b)		✓		✓			✓		✓			
Buckingham and Goodale (2010a)				✓					✓			
Buckingham et al. (2011)				✓					✓			
Buckingham et al. (2012)							✓		✓			
Buckingham and Goodale (2013)				✓	✓				✓			
Buckingham et al. (2014)				✓								
Buckingham et al. (2015)						✓					✓	
Buckingham et al. (2016)				✓		✓			✓		✓	
Buckingham et al. 2018b, a				✓					✓			
Buckingham et al. (2018a, b)				✓					✓			
Chang et al. (2008)		✓		✓			✓		✓			
Chouinard et al. (2009)		✓		✓			✓		✓			
Engel et al. (2020)							✓	✓				
Flanagan and Beltzner (2000)		✓		✓			✓		✓			
Flanagan et al. (2001)		✓		✓			✓		✓			
Flanagan et al. (2008)					✓							
Gordon et al. (1991)		✓		✓			✓		✓			✓
Grandy and Westwood (2006)		✓		✓			✓		✓			
Green et al. (2010)		✓		✓			✓		✓			
Li et al. (2007)							✓					
Li et al. (2011)		✓		✓			✓		✓			✓
Mon-Williams and Murray (2000)		✓	✓				✓	✓				
Naylor et al. (2020)				✓					✓			
Platkiewicz and Hayward (2014)				✓					✓			✓
Rabe et al. (2009)				✓			✓	✓				✓
Rohrbach et al. (2021)				✓					✓			
Saccone et al. (2019a)		✓		✓			✓		✓			
Trewartha and Flanagan (2017)					✓							
van Polanen et al. (2022)					✓					✓		
Total	1	11	1	22	5	2	15	3	20	1	2	4
%	3.3	36.7	3.3	73.3	16.7	6.7	50	10	66.7	3.3	6.7	13.3

^3^ here.

A total of 30 papers are included in the table using 11+ different specific measures. Load force measures include: load force (LF), load force peak (LFP), load force rate (LFR), load force rate peak (LFRP), load force at first peak in load force rate (LF_1st_), and load force rate peak difference (LFRP_Diff_). Grip force measures include: grip force peak (GFP), grip force rate (GFR), grip force rate peak (GFRP), grip force at first peak of grip force rate (GF_1st_), and grip force rate peak difference (GFRP_Diff_). ‘Other’ measures include: vertical acceleration, vertical acceleration peak, and time-course of load force. As can be seen, the most common measures were LFRP, being used in 73.3% of papers, followed by GFRP at 66.7%, GFP at 50%, and LFP at 36.7%.

## Footnotes

1 We define sensorimotor memory as a memory trace associated with a recent sensorimotor experience, such as lifting an object with a given mass, that will influence subsequent motor behaviour, such as affecting lifting forces applied in a second lift. A possible neurological basis for sensorimotor memory has been evaluated by Chouinard et al. (2005).

2 Three participants consistently provided the same magnitude estimate for the light cube in experiment 1. In these cases, we assigned a value of zero for their correlation coefficient.
