# Supplementary material for: New evidence for the sensorimotor mismatch theory of weight perception and the size-weight illusion
Source: Exp Brain Res. 2024 May 23;242(7):1623–43. doi: 10.1007/s00221-024-06849-0 (PMC11208202; doi:10.1007/s00221-024-06849-0)
Supplement: Supplementary file 1 — Supplementary file1 (DOCX 16 kb) [file 221_2024_6849_MOESM1_ESM.docx]

**Supplemental material**

This additional testing was prompted by the discrepant findings in correlations between experiments 1 and 2. Specifically, in experiment 1, the correlations between aggregate forces and perceptual weight estimates for the light and heavy objects were non-significant, while in experiment 2, the correlation between aggregate forces and perceptual weight estimates was significant. One possibility could be that the unexpected changes in mass in experiment 1 and cube size in experiment 2 affected aggregated forces to different extents. Specifically, we wondered whether the effects that applied forces could exert on weight perception were smaller in experiment 1. If this is the case, then the correlations in experiment 1 would be less powerful than those in experiment 2.

To examine this possibility, we calculated difference scores reflecting influences that the switch and size manipulations had on the aggregate force measure. For the switch manipulations, we subtracted the no-switch from the switch aggregate force for the heavy, light, small, and large cubes, producing four difference scores: ΔHeavy (i.e., heavy-after-heavy minus heavy-after-light), ΔLight (i.e., light-after-light minus light-after-heavy), ΔSmall (i.e., small-after-small minus small-after-large), and ΔLarge (i.e., large-after-large minus large-after-small). For the size manipulation, we first calculated the average aggregate force for each cube in experiment 2 (i.e., (small-after-small + small-after-large) /2, and (heavy-after-heavy + heavy-after-small) /2) and then subtracted the average aggregate force for the small from the large cube, producing one difference score: ΔSize.

Afterwards, we performed ANOVA with Difference Score as a within-subject factor (ΔHeavy vs. ΔLight vs. ΔSmall vs. ΔLarge vs. ΔSize). The ANOVA was significant, *F*(4, 555) = 13.8, *p* < .001, *η_p_^2^* = .08 (Greenhouse-Geisser corrected). Bonferroni post hoc testing revealed that there were no differences between ΔHeavy, ΔLight, ΔSmall, and ΔLarge (all *p*s ≥ .999) (Supplementary Table 1). However, ΔSize was significantly higher than all the other difference scores (all *p*s < .001; Supplementary Table 1). In other words, the different sized cubes in experiment 2 caused a greater change in lifting forces than the switching between cubes in both experiments 1 and 2. Based on these results, we conclude that the effects that applied forces could exert on weight perception were smaller in experiment 1.

Supplementary Table 1

| Comparison | | *ΔMean* | *t*(169) | *d* | *p* |
| --- | --- | --- | --- | --- | --- |
| ΔSize | ΔHeavy | 0.30 | 5.81 | 0.61 | < .001* |
|  | ΔLight | 0.28 | 5.43 | 0.57 | < .001* |
|  | ΔSmall | 0.32 | 6.14 | 0.64 | < .001* |
|  | ΔLarge | 0.31 | 6.02 | 0.63 | < .001* |
| ΔHeavy | ΔLight | -0.02 | -0.38 | -0.04 | > .999 |
|  | ΔSmall | 0.02 | 0.34 | 0.04 | > .999 |
|  | ΔLarge | 0.01 | 0.21 | 0.02 | > .999 |
| ΔLight | ΔSmall | 0.04 | 0.71 | 0.07 | > .999 |
|  | ΔLarge | 0.03 | 0.59 | 0.06 | > .999 |
| ΔSmall | ΔLarge | -0.01 | -0.12 | -0.01 | > .999 |

Asterisks (*) denote significant differences after Bonferroni correction (*p* < .05)
